# Impact of body-mass factors on setup displacement in patients with head and neck cancer treated with radiotherapy using daily on-line image guidance

**DOI:** 10.1186/1748-717X-9-19

**Published:** 2014-01-10

**Authors:** Yo-Liang Lai, Shih-Neng Yang, Ji-An Liang, Yao-Ching Wang, Chun-Yen Yu, Ching-Hsiung Su, Shang-Wen Chen

**Affiliations:** 1 Department of Radiation Oncology, China Medical University Hospital, Taichung, Taiwan; 2 School of Medicine, China Medical University, Taichung, Taiwan; 3 School of Medicine, Taipei Medical University, Taipei, Taiwan; 4 Department of Biomedical Imaging and Radiological Science, China Medical University, Taichung, Taiwan

**Keywords:** Adaptive radiotherapy, Body-related factors, Head and neck cancer, Image-guided radiotherapy, Setup error

## Abstract

**Background:**

To determine the impact of body-mass factors (BMF) before radiotherapy and changes during radiotherapy on the magnitude of setup displacement in patients with head and neck cancer (HNC).

**Methods:**

The clinical data of 30 patients with HNC was analyzed using the alignment data from daily on-line on-board imaging from image-guided radiotherapy. BMFs included body weight, body height, and the circumference and bilateral thickness of the neck. Changes in the BMFs during treatment were retrieved from cone beam computed tomography at the 10th and 20th fractions. Setup errors for each patient were assessed by systematic error (SE) and random error (RE) through the superior-inferior (SI), anterior-posterior (AP), and medial-lateral (ML) directions, and couch rotation (CR). Using the median values of the BMFs as a cutoff, the impact of the factors on the magnitude of displacement was assessed by the Mann–Whitney U test.

**Results:**

A higher body weight before radiotherapy correlated with a greater AP-SE (*p* = 0.045), SI-RE (*p* = 0.023), and CR-SE (*p* = 0.033). A longer body height was associated with a greater SI-RE (*p* = 0.002). A performance status score of 1 or 2 was related to a greater AP-SE (*p* = 0.043), AP-RE (*p* = 0.015), and SI-RE (*p* = 0.043). Among the ratios of the BMFs during radiotherapy, the values at the level of mastoid tip at the 20^th^ fraction were associated with greater setup errors.

**Conclusions:**

To reduce setup errors in patients with HNC receiving RT, the use of on-line image-guided radiotherapy is recommended for patients with a large body weight or height, and a performance status score of 1–2. In addition, adaptive planning should be considered for those who have a large reduction ratio in the circumference (<1) and thickness (<0.94) over the level of the mastoid tip during the 20^th^ fraction of treatment.

## Background

Radiation therapy (RT) is commonly used as part of multiple modality treatment for hand and neck cancer (HNC). Intensity-modulated radiation therapy (IMRT) has become increasingly popular because dose escalation to the target can be done while sparing adjacent normal tissues [1]. However, precise patient setup is essential for safe and accurate delivery because IMRT results in steep dose gradients. In HNCs, several factors such as the accuracy of the immobilization device, change in body contours, and tumor regression could lead to setup uncertainties during RT. Causes might be independent or related to the others. Nonetheless, all of these factors need to be minimized with the use of special approaches. Image-guided radiation therapy (IGRT) can be used to correct and quantify geometrical uncertainties for daily setup [2]. Although setup variations can be reduced when using on-line IGRT daily [3,4], the widespread use of daily IGRT is not always feasible in all clinical settings. Adaptive radiotherapy (ART) is an approach used to correct for anatomic changes caused by tumor shrinkage or body weight loss during RT [5]. However, increased medical costs, higher staff workload, and higher radiation doses to the patients are inevitable. As a result, there is a need to investigate suitable indications for ART for patients with HNCs.

There are few studies reporting on setup displacement due to patient-related factors before and during RT for HNC. The main patient-related factors are body-mass factors (BMF) because these affect the stability of immobilization. In addition, correlation between these BMFs and quantification of setup errors remain to be clarified. This study was done to determine the impact of BMFs before and during RT on positioning displacement for patients treated for HNCs. We tried to find correlations between BMFs and the magnitude of daily setup errors. The results can help physicians determine who should be considered for on-line IGRT before starting RT, and select those who need ART to reduce setup displacement.

## Methods

### Patients

With the approval of the local institutional review board, clinical and image data from a cohort of 30 patients treated for HNCs between June 2008 and January 2013 at China Medical University Hospital were reviewed. Twenty-one patients were treated for nasopharyngeal carcinoma, 8 for oropharyngeal cancer, and 1 for hypopharyngeal cancer. Their median age was 53 years (range: 33–77). All patients received IGRT with daily on-line kilovoltage imaging with weekly cone beam computed tomography (CBCT) to correct the treatment position. No patients had ART planning before a prescribed dose of 50 Gy. The characteristics of these patients are listed in Table 1.

**Table 1:** Patient characteristics before radiotherapy

	
Age (y)	
Median (range)	53 (30–77)
Body weight (kilogram)	
Mean (range)	66.9 (41–90.3)
Median	65
Body height (cm)	
Mean (range)	166.2 (153–177)
Median	167.2
BMI	
Mean (range)	24.1 (17.5-29.1)
Median	23.8
ECOG performance status	
0	17
1-2	13
CCRT/RT alone (number)	
CCRT	28
RT alone	2
Tumor origin (number)	
Nasopharynx	21
Oropharynx	8
Hypopharynx	1
Circumference Al (cm)	
Mean ± SD (range)	55.2 ± 3.7 (48.1-62.9)
Thickness A (cm)	
Mean ± SD (range)	16.3 ± 1.3 (14.2-17.9)
Circumference B (cm)	
Mean ± SD (range)	48.4 ± 3.8 (42.2-57.2)
Thickness B (cm)	
Mean ± SD (range)	14.5 ± 1.5 (11.8-18.4)
Circumference C (cm)	
Mean ± SD (range)	41.9 ± 4.5 (33.7-50.6)
Thickness C (cm)	
Mean ± SD (range)	13.6 ± 1.8 (11.0-17.6)

*Abbreviation*: BMI = body mass index; ECOG = Eastern Cooperation Oncology Group; CCRT = concurrent chemoradiotherapy; SD = standard deviation.

Note: Level A was labeled on the mastoid tip, the same section as the junction between the skull base and the 1st cervical vertebra. Level B was based on the mandible angle, the same height as the junction between the 2nd to the 3rd cervical vertebrae. Level C was drawn from the thyroid notch, the same as the 5th cervical vertebra.

### Treatment planning

To enhance the accuracy of the daily irradiated position, all patients were immobilized by a thermoplastic mask (U-shaped Head and Neck Mask, Renfu Medical Equipment, Guangzhou, China) from the bottom of the orbit to the shoulder. Following fabrication of the immobilization device, simulation using a computed tomographic (CT) scan simulator (HiSpeed NX/i, GE Healthcare, Milwaukee, Wisconsin, USA) was made. The scans consisted of a protocol with a 3-mm-slice thickness, and were obtained from the upper orbit to 2 cm below the sternum. Marks on the patients’ skin were drawn using setup lasers to facilitate an accurate daily position.

For patients receiving definitive RT, the clinical target volume (CTV) was defined as the gross tumor volume plus a margin of 1.0 to 1.5 cm. We followed the guidelines for the delineation of an elective nodal CTV [6]. The planning target volume (PTV) was extended 3 mm from the CTV to account for treatment uncertainty. All patients underwent IMRT plans consisting of 7 coplanar fields using 6-MV photons. The prescription dose to the CTV was 50 Gy in 25 fractions followed by a boost to 70–72 Gy to high-risk regions (tumor and involved lymph nodes). All plans were carried out using a commercial radiation treatment planning system (Eclipse version 8.6, Varian Medical Systems Inc, Palo Alto, California, USA).

### Treatment verification

All patients were treated with IGRT with a Varian Clinac iX linear accelerator (Varian Medical Systems, Palo Alto, California, USA) equipped with an on-line On- Board Imaging (OBI) function including two-dimensional (2D) kilovoltage (kV) images and three-dimensional (3D) CBCT. The technicians set up the patients on a couch in the simulation room according to the marks drawn on their bodies. On-line OBI images (2D kV images daily and 3D CBCT weekly) were taken and sent to the station where they could be registered to digitally reconstructed radiographs from the treatment planning images. Two technicians compared these paired images by correlating the bony anatomy and corrected the difference by shifting the couch translationally before treatment. Then, an attending physician confirmed the corrected on-line images. Anatomic reference landmarks included at least three visible bony structures, the vertebra of the C-spine, nasal septum, and mandible profile.

### Anthropometric measures of body-related factors

**Figure 1:**
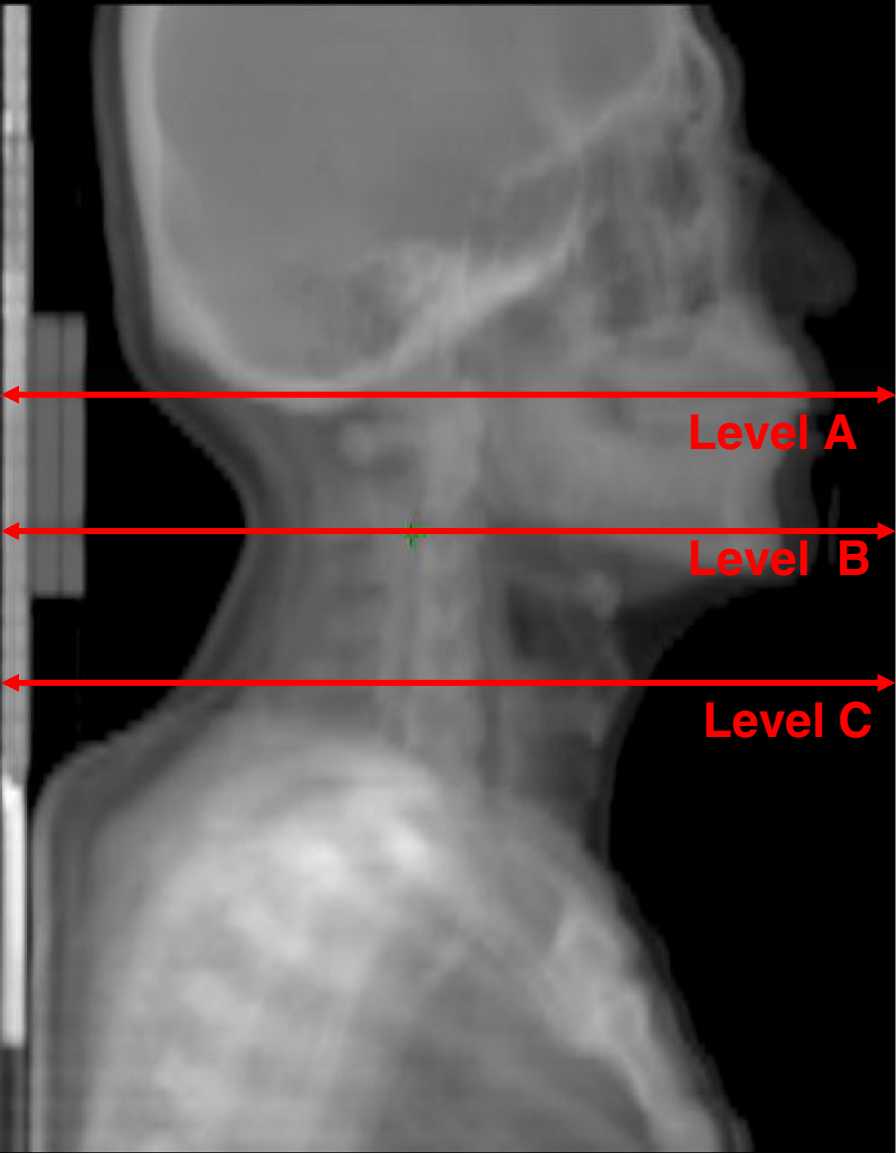
**Circumference and thickness measured across three specified sections.** Level A is labeled on the mastoid tip, the same section as the junction between the skull base and 1st cervical vertebra. Level B is based on the mandible angle, the same height as the junction between the 2nd to the 3rd cervical vertabra. Level C is drawn from the thyroid notch, the same as the 5th cervical vertebra.

Patient-related factors consisted of performance status (PS), age, and BMFs. PS was scored according to the Eastern Cooperative Oncology Group. The BMFs included body weight, body height, body mass index (BMI), and circumference and thickness across three specified sections of the head and neck (Figure 1). Level A was labeled on the mastoid tip, the same section as the junction between the skull base and the 1^st^ cervical vertebra. Level B was based on the mandible angle, the same height as the junction between the 2^nd^ to the 3^rd^ cervical vertebrae. Level C was drawn from the thyroid notch, the same as the 5th cervical vertebra.

BMFs were recorded before RT and at the 10^th^ and 20^th^ fractions during the RT course. Because the circumferences and thicknesses at the three specified levels could not be measured prospectively, the values were retrieved retrospectively according to the CT simulation images and CBCT images during treatment. For circumferences, we first brushed the body contour at a specified section with a fixed thickness to generate a volume. Then, the calculated volumes were divided by the contouring thickness to obtain get the values of the circumferences. The thickness was measured as the maximal transverse distance at the same section as the circumferences. The BMI was calculated as weight in kilograms divided by the square of height in m according to the definition of the World Health Organization.

Patients were monitored weekly and toxicities were recorded according to the Common Terminology Criteria for Adverse Events version 3.0. Ratios of the BMFs during RT were calculated on the three levels. For example, the ratio of the circumference on Section A at the 10^th^ treatment was the circumference at level A at the 10^th^ fraction divided by the pretreatment circumference.

### Setup displacement

After image registration, quantification of alignment data for daily OBI in the superior-inferior (SI), anterior-posterior (AP), and medial-lateral (ML) directions, and couch rotation (CR) for all patients were collected. For each direction, the recorded setup displacements were composed of two components, systematic errors (SE) and random errors (RE). The SE was the deviation between the simulated patient position and the average patient position, while the RE was that which occurred between different fractions. The detailed calculation for SE and RE was similar to that used by Remeijer et al. [7].

By analyzing all the alignment data before the 25 fractions of treatment for each patient, the values of SE and RE for all directions in each patient could be calculated.

### Statistical analysis

We used the Mann–Whitney U test to determine the correlation between the magnitude of errors and patient-related factors. The median values of the BMFs were used as a cut-off to divide lower and higher groups. We also used the Pearson correlation coefficient to examine the association between the reduction ratio of body weight and circumferences or thicknesses. In addition, the same test was used to examine the relation across the displacement in different directions. A two-sided *p* value of < 0.05 was considered statistically significant. All statistical analyses were performed using a commercial software package (SPSS 13.0 for Windows, Chicago, IL, USA).

## Results

### Baseline clinical characteristics

The median values for various BMFs are listed in Table 1. The mean displacements in the SI, AP, and ML directions for all patients were 1.3 mm, 1.6 mm, and 2.2 mm, respectively. The population SE was, 2.2 mm, 1.1 mm, and 1.6 mm and the RE, 1.5 mm, 0.9 mm and 1.1 mm for the SI, AP and ML directions, respectively. Van Herk et al. [8] suggested a CTV- PTV margin of 2.5 SE + 0.7 RE to ensure that 90% of patients in a population receive a minimum cumulative CTV dose of at least 95% of the prescribed dose. Under this definition, the suggested CTV-PTV margins for setup uncertainties in our patients were 3.4, 4.8 and 6.5 mm in the AP, ML and SI directions, respectively. The population SE and RE for CR were 0.31 and 0.36 degrees, respectively.

There was no correlation across the displacement among the three translation directions. However, the SE and RE for the CR showed an association with the ML-SE (r = 0.49, *p* = 0.007 and r =0.44, *p* = 0.016).

**Figure 2:**
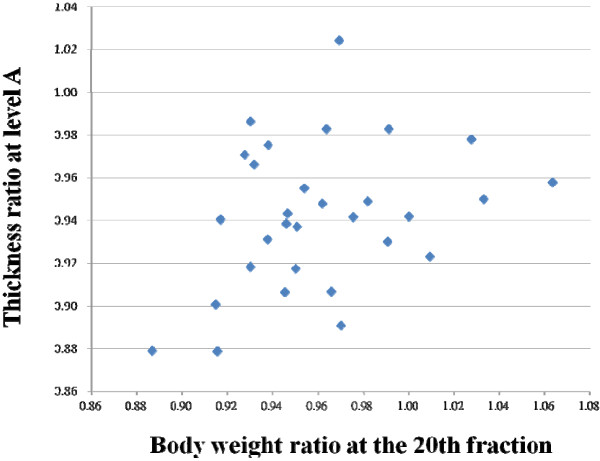
**The association between the ratio of body weight and thickness at level A during the 20th fraction of cone beam computed tomography (r = 0.32, *p*** **= 0.081).**

We found the correlation was not significant between the ratio of body weight and thickness at level A on the CBCT at the 20^th^ fraction (r = 0.32, *p* = 0.081), which is depicted in Figure 2. In addition, there was no correlation between the ratio of body weight and circumference at any level either at the 10^th^ or 20^th^ fraction.

### Correlation between pretreatment body-mass factors and setup displacement

Table 2 summarizes the association between the mean values of setup displacements in the three translational directions and the BMFs. Of the pretreatment factors, a larger body weight was significantly correlated with a greater AP-SE (*p* = 0.045) and SI-RE (*p* = 0.023), and a longer body height was associated with a greater SI-RE (*p* = 0.002). A PS score of 1 or 2 was associated with a greater AP-SE (*p* = 0.043), AP-RE (*p* = 0.015), and SI-RE (*p* = 0.043).

**Table 2:** Setup displacement (mean ± standard deviation in mm) in three translational directions according to lower and higher 50% percentile of body-related factors before RT

**Variable**	**AP-SE**	**AP-RE**	**SI-SE**	**SI-RE**	**ML-SE**	**ML-RE**
Body weight						
Lower	0.9 ± 0.5	0.8 ± 0.3	2.1 ± 0.9	1.2 ± 0.4	1.8 ± 0.8	1.1 ± 0.3
Higher	1.3 ± 0.6	0.9 ± 0.3	2.4 ± 1.0	1.8 ± 0.9	1.9 ± 0.6	1.0 ± 0.4
*p* value	0.045*	0.250	0.567	0.023*	0.436	0.461
Body height						
Lower	1.0 ± 0.6	0.8 ± 0.3	1.9 ± 0.6	1.1 ± 0.3	1.8 ± 0.7	1.1 ± 0.3
Higher	1.3 ± 0.5	0.9 ± 0.3	2.6 ± 1.1	1.9 ± 0.9	1.9 ± 0.7	1.1 ± 0.4
*p* value	0.081	0.161	0.116	0.002*	0.250	0.838
BMI						
Lower	1.0 ± 0.5	0.8 ± 0.3	2.2 ± 0.9	1.4 ± 0.7	1.7 ± 0.8	1.1 ± 0.3
Higher	1.3 ± 0.6	0.9 ± 0.3	2.3 ± 1.1	1.6 ± 0.8	2.0 ± 0.5	1.1 ± 0.4
*p* value	0.187	0.512	0.935	0.217	0.305	0.935
Circumference A						
Lower	1.0 ± 0.5	0.8 ± 0.2	2.1 ± 1.0	1.4 ± 0.7	1.8 ± 0.8	1.1 ± 0.4
Higher	1.3 ± 0.6	0.9 ± 0.3	2.3 ± 1.0	1.6 ± 0.8	1.9 ± 0.6	1.0 ± 0.3
*p* value	0.345	0.345	0.775	0.461	0.624	0.267
Circumference B						
Lower	1.0 ± 0.5	0.8 ± 0.3	2.1 ± 1.0	1.4 ± 0.7	1.7 ± 0.8	1.1 ± 0.4
Higher	1.3 ± 0.6	0.8 ± 0.3	2.4 ± 1.0	1.6 ± 0.8	2.0 ± 0.5	1.0 ± 0.3
*p* value	0.389	0.486	0.305	0.187	0.345	0.412
Circumference C						
Lower	1.1 ± 0.5	0.8 ± 0.3	2.3 ± 1.1	1.4 ± 0.7	1.7 ± 0.8	1.0 ± 0.4
Higher	1.2 ± 0.6	0.9 ± 0.3	2.2 ± 0.8	1.6 ± 0.8	2.0 ± 0.5	1.1 ± 0.3
*p* value	0.486	0.567	0.967	0.345	0.250	0.683
PS						
0	0.9 ± 0.5	0.7 ± 0.2	2.0 ± 0.7	1.2 ± 0.4	2.0 ± 0.6	1.1 ± 0.4
1-2	1.4 ± 0.6	1.0 ± 0.3	2.6 ±1.2	1.9 ± 1.0	1.6 ± 0.7	1.0 ± 0.4
*p* value	0.043*	0.015*	0.245	0.043*	0.072*	0.934

*Abbreviation*: AP = anterior-posterior, SI = superior-inferior, ML = medial-lateral, SE = systemic error, RE = random error, BMI = body mass index, PS = performance status.

Note: asterisk represents statistical significance.

In addition, body height was associated with the CR-SE (*p* = 0.033) and marginally correlated with the CR-RE (*p* = 0.067). For patients with longer and shorter body heights, the mean CR-SE was 0.22 ± 0.21 and 0.40 ± 0.25 degrees, respectively. The other pretreatment factors such as age, and BMI, were not associated with the magnitude of setup errors.

### Correlation between reduction ratio of body-mass factors during RT and setup displacement

The alignment data before the CBCT of the 20th fraction were included when investigating the correlation between the setup errors and the change of the BMFs. Among the ratios of the BMFs during RT, the values at each level were divided into lower and higher 50%. Compared with the ratios of body weight, our result showed certain BMFs can be more useful for initiating ART. At the 10th fraction of CBCT, the ratio of the thickness at level A was correlated with a greater ML-RE (*p* = 0.043), whereas the ratio of the thickness at level B was associated with a greater ML-RE (*p* = 0.019) (Table 3). At the 20th fraction, the ratio of the circumference at level A had a significant correlation with a greater AP-SE (*p* = 0.019), AP-RE (*p* = 0.019), and SI-SE (*p* = 0.025). The ratio of the thickness at level A was significantly correlated with a greater ML-SE (*p* =0.013), whereas it showed a marginal impact with the SI-RE (*p* =0.05) (Table 4). The remaining ratio of the BMFs had no impact on setup errors (Additional file 1).

**Table 3:** Setup displacement (mean ± standard deviation in mm) in three translational directions according to lower and higher 50% percentile of body-related factors during RT (10th fraction)

**Variable**	**AP-SE**	**AP-RE**	**SI-SE**	**SI-RE**	**ML-SE**	**ML-RE**
rT (level A)						
Lower (<0.98)	1.0 ± 0.6	0.8 ± 0.3	2.3 ± 1.2	1.6 ± 0.8	2.0 ± 0.6	1.2 ± 0.4
Higher (>0.98)	1.2 ± 0.5	0.9 ± 0.3	2.1 ± 0.8	1.4 ± 0.7	1.7 ± 0.7	1.0 ± 0.3
*p* value	0.193	0.294	0.854	0.423	0.224	0.043*
rT (level B)						
Lower (<0.97)	1.1 ± 0.6	0.8 ± 0.2	2.2 ± 1.1	1.3 ± 0.5	1.8 ± 0.7	1.2 ± 0.4
Higher (>0.97)	1.2 ± 0.6	0.9 ± 0.3	2.3 ± 0.9	1.6 ± 1.0	1.9 ± 0.7	0.9 ± 0.3
*p* value	0.667	0.918	0.473	0.448	0.334	0.019*
rBW						
Lower (<0.99)	1.2 ± 0.6	0.9 ± 0.2	2.2 ± 0.9	1.4 ± 0.5	2.0 ± 0.8	1.2 ± 0.4
Higher (>0.99)	1.1 ± 0.5	0.9 ± 0.3	2.2 ± 1.1	1.5 ± 0.9	1.8 ± 0.6	1.0 ± 0.3
*p* value	0.851	0.884	0.819	0.884	0.787	0.267

*Abbreviation*: rT = ratio of thickness, during-RT/pre-RT; rBW = ratio of body weight, during-RT/pre-RT; AP = anterior-posterior, SI = superior-inferior; ML = medial-lateral; SE = systematic error; RE = random error.

Note: asterisk respresents statistical significance.

**Table 4:** Setup displacement (mean ± standard deviation in mm) in three translational directions according to lower and higher 50% percentile of body-related factors during RT (20th fraction)

**Variable**	**AP-SE**	**AP-RE**	**SI-SE**	**SI-RE**	**ML-SE**	**ML-RE**
rC (level A)						
Lower (<1)	1.4 ± 0.5	1.0 ± 0.2	2.5 ± 0.9	1.8 ± 0.9	1.6 ± 0.7	1.0 ± 0.3
Higher (>1)	0.9 ± 0.5	0.7 ± 0.2	2.0 ± 1.0	1.2 ± 0.5	2.0 ± 0.6	1.1 ± 0.4
*p* value	0.019*	0.019*	0.093	0.025*	0.101	0.854
rT (level A)						
Lower (<0.94)	1.3 ± 0.6	0.9 ± 0.3	2.5 ± 1.2	1.7 ± 0.8	2.2 ± 0.6	1.1 ± 0.3
Higher (>0.94)	1.0 ± 0.5	0.8 ± 0.2	2.0 ± 0.6	1.3 ± 0.7	1.5 ± 0.6	1.0 ± 0.4
*p* value	0.325	0.325	0.461	0.050	0.013*	0.217
rBW						
Lower (<0.95)	1.2 ± 0.5	0.9 ± 0.2	2.5 ± 1.0	1.5 ± 0.5	1.8 ± 0.6	1.1 ± 0.4
Higher (>0.95)	1.1 ± 0.6	0.9 ± 0.3	2.0 ±1.0	1.5 ± 1.0	1.9 ± 0.7	1.0 ± 0.3
*p* value	0.377	0.608	0.193	0.334	0.790	0.608

*Abbreviation*: rC = ratio of circumference, during-RT/pre-RT; rT = ratio of thickness, during-RT/pre-RT; rBW = ratio of body weight, during-RT/pre-RT; AP = anterior-posterior, SI = superior-inferior; ML = medial-lateral; SE = systematic error; RE = random error.

Note: asterisk represents statistical significance.

The ratio of the thickness at level A at the 20^th^ fraction was associated with a greater CR-SE (*p* = 0.009) and CR-RE (*p* = 0.019).

## Discussion

Several studies have investigated setup uncertainty in HNC patients [9-13], which could change the dose distribution to the target volume and organs at risk [9,12]. Certainly, IGRT and ART are two solutions for these limitations [5,14]. Furthermore, according to the International Commission on Radiation Units and Measurements report 62 [15], an inappropriate definition of the CTV-PTV margin, accounting for organ motion and setup uncertainties, may yield an underdose to the CTV. In order to define this margin for HNC, organ motion could be neglected, while variability due to inadequate setup or deformity must be carefully considered [11]. In clinical practice, extensive use of daily IGRT is not always possible because of limited facilities in some countries as well as concerns about increased daily doses to patients [14]. Because there is a lack of evidence regarding patients who are vulnerable to setup uncertainty, we first reported the impact of pretreatment patient-related factors on setup displacement. Based on our findings, physicians can select appropriate patients for IGRT, such as those with a large body weight or height, or a PS of 1 or above. For patients who are not able to have IGRT for certain reasons, a sufficient CTV-PTV margin in a specific direction would be required to minimize uncertainties. Currently in our department, PTVs are generated by adding a 3 mm margin to corresponding CTVs; this study disclosed the inadequacy of this approach in certain directions for those prone to setup uncertainty if withholding IGRT. Because there might be great variety in setup accuracy among institutions and some geographical or racial difference in BMFs [16], an in-house report is imperative to minimize uncertainties. The current study provided a simple approach for this.

For patients receiving RT for HNCs, anatomical modifications due to tumor regression and body weight loss are easily noted during the RT course. Most physicians plan ART according to body weight changes or an unfitted immobilization device. Meanwhile, the geometric change of tumor volume and organs at risk should also be assessed. Currently, guidelines based on scientific data for initiating ART are still lacking. Although it has been suggested that an ART plan not only improves dosimetric benefits but also increases tumor control and quality of life [17-19], it may increase costs to patients and the workload of clinical staff. Our work is a pilot study correlating changes in BMFs and the magnitude of setup displacements in HNC. Accordingly, ART planning can be initiated to minimize uncertainties for patients with a great reduction of the circumference or thickness at the level of mastoid tip at the 20^th^ fraction of RT. Otherwise, widening of the CTV-PTV margin in the three translation directions should be done. For example, the margin in the SI direction could be expanded up to 7.4 mm for those with large changes in BMFs, according to the formula of Van Herk et al. [8].

Among studies of setup analyses according to time trend, Mongioj et al. [10] investigated alignment data from a cohort of 20 patients with nasopharyngeal cancer. They found setup displacements showed no significant changes as therapy progressed, but greater errors were observed when the patient had severe weight loss or tumor node shrinkage. In addition, grade 2 toxicities were associated with great displacement along the AP and SI directions. However, the change of BMFs was not correlated with the setup data. In the future, more studies are essential before these parameters during RT become a reference for initiating ART. In addition, investigations should be done to find the more significant anatomical level when assessing changes in BMFs.

Generally, our study disclosed most patients had a reduction in BMFs with time. However, we found some patients had a ratio of BMFs ≧ 1 during the RT course (shown in Tables 3, 4, and Additional file 1), particularly at the 10th fraction of RT. One plausible reason is that shrinkage of soft tissue after RT might lead to an insufficient immobilization effect from the thermoplastic mask. As a result, the credibility of the calculated values across the three levels of the BMFs might be challenged because of displacement of the patient’s neck. In addition, the effect of intravenous fluid overload on neck swelling during concurrent administration of chemotherapy should be investigated further. To circumvent the limitations of the study, daily CBCT should be implemented to verify the reproducibility of the BMFs.

Our study should be interpreted with several concerns. First, circumferences and thicknesses were not obtained prospectively by measuring the body with a ruler, but were measured retrospectively from CT images. The absolute values and the ratio of the BMFs might be inconsistent with those acquired from direct measurement. According to previous comparison tests in some patients, the maximal deviation between the two methods was less than 5%. Thus, this approach can be applied in those institutions ready to initiate the analysis. Second, errors due to anatomical changes should be distinguished from those caused by inadequate setup. The problem could be circumvented by the approach reported by Mc Dermott et al. [20]. They suggested using differences in images created by subtracting the first localization image from that of subsequent fractions was an efficient way to qualitatively detect anatomical changes during RT. Third, the median value was taken as a cut-off for each measure to create two groups for comparison. As a result, a lot of different groups were created. To achieve more detailed stratification of the BMFs, there is a need to conduct a study with large sample size. Finally, our study did not report the impact of daily CBCT on setup displacement, as well as the weekly dosimetric changes. Although tumor and soft tissue targets could be assessed by CBCT, Li et al. [2] suggested that there were no statistically significant differences in alignment between 2D kV and 3D CBCT images in HNCs. Thus, the consistency between setup errors for the bony structure and the target could be established. Future studies should enroll more patients prospectively, and evaluate subsequent dosimetric changes according to evolution of the BMFs.

## Conclusions

This study recommends on-line IGRT for patients with HNC receiving RT who have a large body weight or height, or have a PS score of 1–2. In addition, to deliver more accurate dose to tumor and avoid extra dose to organs at risk due to anatomical change, an adaptive planning should be considered for those who have a large reduction ratio in the circumference (< 1) and thickness (< 0.94) over the level of the mastoid tip at the 20^th^ fraction of treatment.

## Competing interests

All authors declare that no actual or potential conflicts of interest were encountered during this study.

## Authors’ contributions

YL Lai, CY Yu, and SW Chen were responsible for design of the study, acquisition of data, analysis and interpretation of data, and drafting the article. SN Yang, JA Liang, and CH SU provided some intellectual content. SW Chen approved the version to be submitted. All authors read and approved the final manuscript.

## Supplementary Material

Additional file 1: Table S1 Setup displacement (mean ± standard deviation in mm) in three translational directions according to lower and higher 50% percentile of body-related factors (level B and C) during RT (10th fraction). **Table S2.** Setup displacement (mean ± standard deviation in mm) in three translational directions according to lower and higher 50% percentile of body-related factors (level B and C) during RT (20th fraction). Click here for file
