# Supplementary material for: Impact of body-mass factors on setup displacement in patients with head and neck cancer treated with radiotherapy using daily on-line image guidance
Source: Radiat Oncol. 2014 Jan 10;9:19. doi: 10.1186/1748-717X-9-19 (PMC3904466; doi:10.1186/1748-717X-9-19)
Supplement: Additional file 1: Table S1 — Setup displacement (mean ± standard deviation in mm) in three translational directions according to lower and higher 50% percentile of body-related factors (level B and C) during RT (10th fraction). Table S2. Setup displacement (mean ± standard deviation in mm) in three translational directions according to lower and higher 50% percentile of body-related factors (level B and C) during RT (20th fraction). [file 1748-717X-9-19-S1.doc]

**Additional file**

Table S1. Setup displacement (mean ± standard deviation in mm) in three translational directions according to lower and higher 50% percentile of body-related factors (level B and C) during RT (10^th^ fraction)

| Variable | AP-SE | AP-RE | SI-SE | SI-RE | ML-SE | ML-RE |
| --- | --- | --- | --- | --- | --- | --- |
| rC(level A)  Lower (<1.0)  Higher (>1.0)  *p* value | 1.3 ± 0.7  1.0 ± 0.5  0.423 | 0.9 ± 0.3  0.8 ± 0.2  0.377 | 2.4 ± 0.9  2.1 ± 1.0  0.423 | 1.5 ± 0.6  1.5 ± 0.9  0.275 | 1.8 ± 0.9  1.9 ± 0.5  0.822 | 1.0 ± 0.2  1.1 ± 0.4  0.637 |
| rC(level B) |  |  |  |  |  |  |
| Lower (<1.0)  Higher (>1.0)  *p* value | 1.2 ± 0.7  1.1 ± 0.5  0.902 | 0.9 ± 0.3  0.9 ± 0.2  1.000 | 2.0 ± 0.6  2.4 ± 1.1  0.245 | 1.5 ± 0.8  1.5 ± 0.7  0.536 | 2.0 ± 0.7  1.8 ± 0.7  0.509 | 1.1 ± 0.4  1.0 ± 0.4  0.229 |
| rC(level C) |  |  |  |  |  |  |
| Lower (<1.0)  Higher (>1.0)  *p* value | 1.1 ± 0.5  1.3 ± 0.6  0.346 | 0.8 ± 0.3  0.9 ± 0.3  0.172 | 2.2 ± 1.0  2.2 ± 1.0  0.983 | 1.5 ± 0.7  1.5 ± 0.8  0.950 | 1.8 ± 0.7  1.9 ± 0.7  0.518 | 1.0 ± 0.3  1.1 ± 0.4  0.491 |
| rT(level C) |  |  |  |  |  |  |
| Lower (<0.98)  Higher (>0.98)  *p* value | 1.1 ± 0.5  1.2 ± 0.6  0.653 | 0.8 ± 0.2  0.9 ± 0.3  0.683 | 2.2 ± 0.9  2.3 ± 1.1  0.838 | 1.7 ± 0.9  1.3 ± 0.5  0.325 | 1.9 ± 0.8  1.9 ± 0.5  0.838 | 1.1 ± 0.4  1.0 ± 0.2  0.744 |

Abbreviation: rC = ratio of circumference, during-RT / pre-RT; rT = ratio of thickness, during-RT / pre-RT; AP = anterior-posterior, SI = superior-inferior; ML = medial-lateral; SE = systematic error; RE = random error.

Note: asterisk represents statistical significance.

Table S2. Setup displacement (mean ± standard deviation in mm) in three translational directions according to lower and higher 50% percentile of body-related factors (level B and C) during RT (20^th^ fraction)

| Variable | AP-SE | AP-RE | SI-SE | SI-RE | ML-SE | ML-RE |
| --- | --- | --- | --- | --- | --- | --- |
| rC(level B) |  |  |  |  |  |  |
| Lower (<1.0)  Higher (>1.0)  *p* value | 1.3 ± 0.6  1.0 ± 0.5  0.202 | 0.9 ± 0.3  0.8 ± 0.2  0.653 | 2.2 ± 0.9  2.3 ± 1.1  0.744 | 1.6 ± 0.7  1.4 ± 0.8  0.174 | 1.9 ± 0.5  1.9 ± 0.8  0.838 | 1.1 ± 0.4  1.0 ± 0.4  0.683 |
| rT(level B)  Lower (<0.94)  Higher (>0.94)  P value | 1.2 ± 0.6  1.1 ± 0.5  0.436 | 0.9 ± 0.3  0.8 ± 0.3  0.436 | 2.2 ± 1.1  2.2 ± 1.0  0.713 | 1.6 ± 0.8  1.4 ± 0.7  0.486 | 2.0 ± 0.6  1.8 ± 0.7  0.345 | 1.1 ± 0.4  1.1 ± 0.4  0.902 |
| rC(level C) |  |  |  |  |  |  |
| Lower (<0.98)  Higher (>0.98)  *p* value | 1.1 ± 0.5  1.2 ± 0.6  1.000 | 0.8 ± 0.2  0.9 ± 0.3  0.202 | 2.3 ± 0.9  2.2 ±1.1  0.595 | 1.6 ± 0.7  1.3 ± 0.7  0.137 | 1.9 ± 0.6  1.8 ± 0.7  0.902 | 1.0 ± 0.4  1.1 ± 0.3  0.567 |
| rT(level C) |  |  |  |  |  |  |
| Lower (<0.94)  Higher (>0.94)  *p* value | 1.2 ± 0.5  1.1 ± 0.6  0.624 | 0.8 ± 0.2  0.9 ± 0.3  0.683 | 2.2 ± 0.9  2.2 ± 1.1  0.838 | 1.5 ± 0.8  1.5 ± 0.7  0.935 | 1.9 ± 0.7  1.8 ± 0.7  0.683 | 1.0 ± 0.4  1.1 ± 0.4  0.744 |

Abbreviation: rC = ratio of circumference, during-RT / pre-RT; rT = ratio of thickness, during-RT / pre-RT; AP = anterior-posterior, SI = superior-inferior; ML = medial-lateral; SE = systematic error; RE = random error.

Note: asterisk represents statistical significance.
